# Direct test of the FLRW metric from strongly lensed gravitational wave observations

**DOI:** 10.1038/s41598-019-47616-4

**Published:** 2019-08-12

**Authors:** Shuo Cao, Jingzhao Qi, Zhoujian Cao, Marek Biesiada, Jin Li, Yu Pan, Zong-Hong Zhu

**Affiliations:** 1 Department of Astronomy, Beijing Normal University, Beijing, 100875 China; 2 Department of Physics, College of Sciences, Northeastern University, 110819 Shenyang, China; 3 Department of Astrophysics and Cosmology, Institute of Physics, University of Silesia, 75 Pułku Piechoty 1, 41-500 Chorzów, Poland; 4 Department of Physics, Chongqing University, Chongqing, 400030 China; 5 College of Science, Chongqing University of Posts and Telecommunications, Chongqing, 400065 China

**Keywords:** Cosmology, Galaxies and clusters

## Abstract

The assumptions of large-scale homogeneity and isotropy underly the familiar Friedmann-Lemaître-Robertson-Walker (FLRW) metric that appears to be an accurate description of our Universe. In this paper, we propose a new strategy of testing the validity of the FLRW metric, based on the galactic-scale lensing systems where strongly lensed gravitational waves and their electromagnetic counterparts can be simultaneously detected. Each strong lensing system creates opportunity to infer the curvature parameter of the Universe. Consequently, combined analysis of many such systems will provide a model-independent tool to test the validity of the FLRW metric. Our study demonstrates that the third-generation ground based GW detectors, like the Einstein Telescope (ET) and space-based detectors, like the Big Bang Observer (BBO), are promising concerning determination of the curvature parameter or possible detection of deviation from the FLRW metric. Such accurate measurements of the FLRW metric can become a milestone in precision GW cosmology.

## Introduction

It is well known that Friedmann–Lemaître–Robertson–Walker (FLRW) metric, an exact solution of the Einstein’s equations obtained under the assumption of homogeneity and isotropy of space, is very successful in explaining many observational facts concerning our Universe including large-scale distribution of galaxies and the near-uniformity of the CMB temperature^1^. A particularly successful application of the FLRW metric is that it underlies the present standard cosmological model, which is very successful in fitting the current observational data sets and explaining the observed cosmic acceleration. Significant evidence that the space-time metric in cosmological scales deviates from the FLRW metric would have profound consequences for inflation theory and fundamental physics. For instance, the observed phenomenon of late-time accelerated expansion can be attributed to the failure of the FLRW approximation^2,3^, which motivated the search for alternative solutions including the effect of inhomogeneities in small scales on the average expansion rate^4,5^, as well as the violation of statistical homogeneity and isotropy^6–10^. The last two decades brought rapid advances in observational cosmology, which made it possible to test FLRW metric by checking the consistency between different observables derived from general assumptions of geometrical optics^11–14^. Recently^15^, presented a new test of the validity of the FLRW metric, using the combination of independent observations: Union2.1 supernova distances and galaxy strong lensing data from Sloan Lens ACS Survey. Let us note that the previous works focused on the reconstruction of a smooth distance function with supernova data, which were used to estimate the luminosity distance to lenses and sources^16,17^.

On the other hand, the first direct detection^18^ of the gravitational wave (GW) source GW150914 has opened an era of gravitational wave astronomy and added a new dimension to the multi-messenger astrophysics. Possibility of using GW signal from inspiraling binary system to determine the absolute value of its luminosity distance and thus the Hubble constant were first noticed in^19^. In recent works inspiraling binary black holes (BHs) and neutron stars (NS) have successfully been used as standard sirens^20^. Moreover, the effect of gravitational lensing of GWs has recently gained interest and has been studied extensively. The earliest attempts to discuss lensing of GW signals from merging NS binaries, its influence on the inferred luminosity distance to the source, expected total number of events and their redshift distribution can be traced back to^21^. Some recent works took even more extreme view suggesting that the first six binary BH merging events reported by LIGO/Virgo should be reinterpreted as being lensed and proposing that GW170809 and GW170814 events are the lensed signals from the single source^22^. However, The interest in detecting a lensed GW signal is powered by suggestions that cosmological parameters can be significantly constrained using time delays measurements of strongly lensed GW events^23^. Moreover, strongly lensed GW signals can be used to test fundamental physics. For example, the speed of gravity can be tested with strongly lensed GW events accompanied by electromagnetic counterparts^24^. Recent analysis revealed that detection of lensed events with advanced LIGO detectors could be quite plausible^25^. In the context of third generation interferometric detectors, a series of papers^26^ explored the perspectives of observing gravitationally lensed coalescing double compact objects in the Einstein Telescope (ET). The detailed calculation of GW lensing rate caused by lensing galaxies showed that ET would register about 100 strongly lensed inspiral events per year at its design sensitivity. In this paper, we propose that lensed GW signals accompanied by EM counterpart would enable determination of cosmic curvature and would provide a test of validity of the FLRW metric. The main motivation of this work is that lensed GWs can be probed at much higher redshifts than SNIa or the large scale structure^27^.

Homogeneous and isotropic spacetime is described by the FLRW metric

1 $$d{s}^{2}={c}^{2}d{t}^{2}-\frac{a{(t)}^{2}}{1-K{r}^{2}}d{r}^{2}-a{(t)}^{2}{r}^{2}d{{\rm{\Omega }}}^{2},$$

where *a* represents the scale factor (in the unit of [length]). The dimensionless curvature *K* is related to the cosmic curvature parameter as ${{\rm{\Omega }}}_{k}=-\,K{c}^{2}/{a}_{0}^{2}{H}_{0}^{2}$, where *H*_0_ denotes the Hubble constant (in the unit of [time]^−1^) and *a*_0_ is the present value of the scale factor. Let us introduce dimensionless comoving distances *d*_*ls*_ ≡ *d*(*z*_*l*_, *z*_*s*_), *d*_*l*_ ≡ *d*(0, *z*_*l*_) and *d*_*s*_ ≡ *d*(0, *z*_*s*_), related to the (dimensioned) comoving distances *D* as *d* = *H*_0_*D*/*c*. In the subsequent simulations of strongly lensed GW data, we use the Hubble constant measurement of *H*_0_ = 67.8 ± 0.9 km s^−1^ M*pc*^−1^, determined from Planck 2015 temperature data combined with Planck lensing^1^. In the flat universe, dimensionless comoving distances between the observer and two other aligned objects *l* and *s* will obey an additivity relation *d*_*ls*_ = *d*_*s*_ − *d*_*l*_. In non-flat FLRW models this relation is modified to ${d}_{ls}=\sqrt{1+{{\rm{\Omega }}}_{k}{d}_{l}^{2}}{d}_{s}-\sqrt{1+{{\rm{\Omega }}}_{k}{d}_{s}^{2}}{d}_{l}$^15^. Such setting is realized in Nature by strong gravitational lensing systems, where the source located at redshift *z*_*s*_ and intervening galaxy acting as a lens located at redshift *z*_*l*_ are almost perfectly aligned. Admittedly, we use FLRW metric in order to be specific how to calculate distances in the distance sum rule. However, in our method all necessary distances can be assessed in each strong lensing system directly without tacit assumptions underlying the extrapolation from SN Ia, for example. FLRW metric invoked is just a framework whose consistency is being checked. From the location of multiple images of a strong lensing system, one is able to assess the ratio of angular-diameter distances $\frac{{D}_{ls}^{A}}{{D}_{s}^{A}}=\frac{{d}_{ls}}{{d}_{s}}$ (see the Methods section for details). Therefore, by virtue of the distance sum rule^15^, one is able to measure

2 $$\frac{{d}_{ls}}{{d}_{s}}=\sqrt{1+{{\rm{\Omega }}}_{k}{d}_{l}^{2}}-\frac{{d}_{l}}{{d}_{s}}\sqrt{1+{{\rm{\Omega }}}_{k}{d}_{s}^{2}}.$$

If the other two dimensionless comoving distances *d*_*l*_ and *d*_*s*_ can be measured, then the value of Ω_*k*_ could be determined. As we will discuss in more details in the Methods section, all these necessary ingredients can be derived from multiple information accessible in particular type of lensing systems. In this paper, we focus on galactic-scale strong gravitational lensing systems with high-redshift inspiraling NS-NS and NS-BH binaries acting as background sources. The EM counterpart would allow the host galaxy and lens galaxy identification and state-of-the-art lens modelling techniques would enable a precise reconstruction of lens mass distribution.

One of the typical features of lensed GW signals is that time delays between lensed images (e.g. 1~100 days) inferred from the GW observations would have uncertainties (e.g. ~0.1 *s* from the detection pipeline) totally negligible comparing to the uncertainty in lens modeling, while other relevant observables, like redshifts or images, can be precisely measured in the EM domain. This allows the assessment of the time delay distance *D*_Δ*t*_ and the transverse comoving distance to the lens *D*_*l*_ (see the Methods section for details).

Finally, as already mentioned, GW signals from inspiraling and merging compact binary systems can provide the luminosity distance from the observer to the source ${D}_{s}^{L}$. The lensing amplification *μ* boosts^21^ the amplitude of the GW strain signal *A* by a factor $\sqrt{\mu }$ and accompanying EM flux by a factor *μ*. Once the lens potential is recovered from the image analysis combined with time delays, the magnification factors of images can be estimated. Consequently, the transverse comoving distance *D*_*s*_ can be derived. Proceeding along the steps outlined in the Method section, we find that the Ω_*k*_(*z*_*l*_, *z*_*s*_) values for different pairs (*z*_*l*_, *z*_*s*_) can be directly obtained. Due to the strong covariance between *d*_*l*_, *d*_*s*_, and *d*_*ls*_, instead of propagating distance uncertainties, we use Monte-Carlo simulation to project uncertainties in the lens mass profile, time delays, Fermat potential difference, and the magnification effect onto the final uncertainty of Ω_*k*_(*z*_*l*_, *z*_*s*_). This creates an opportunity to test the validity of the FLRW metric. Namely, in the FLRW universe Ω_*k*_(*z*_*l*_, *z*_*s*_) should be constant and equal to the present value of curvature parameter e.g. inferred from CMB anisotropies. However, if one notices significant differences between Ω_*k*_(*z*_*l*_, *z*_*s*_) values for different pairs (*z*_*l*_, *z*_*s*_), which cannot be accounted for by systematics and scatter, then this could be a signal that FLRW description breaks down. The converse is not true: if Ω_*k*_(*z*_*l*_, *z*_*s*_) is constant, it does not indicate that light propagation on large scales is not described by the FLRW metric^15^.

## Results

Table 1 summarizes the uncertainties of multiple measurements in GW and EM lensing systems. The justification for the values reported is given below.

**Table 1:** Relative uncertainties of respective factors contributing to the accuracy of Ω_*k*_(*z*_*l*_, *z*_*s*_) measurement.

	*δθ*_*E*_	*δσap*	*δγ*
Image configuration	1%	5%	1%
	*δ*Δ*t*	*δ*Δ*ψ*	*δ*Δ*ψ*(*LOS*)
Time delay	0%	~(*δθE*,*δγ*)	1%
	$\delta {d}_{s}^{L}$ (SNR)	$\delta {d}_{s}^{L}$ (WL)	*δF* (SL + ML)
Lensed GW	2/*ρnet*	0.05*z*	10% (50%)

*δθ*_*E*_, *δσ*_*ap*_, and *δγ* denote Einstein radius, aperture velocity dispersion, and mass-density profile; *δ*Δ*t*, *δ*Δ*ψ*, *δ*Δ*ψ*(*LOS*) correspond to time delay, Fermat potential difference and light-of-sight contamination, respectively; $\delta {d}_{s}^{L}$ and *δF* correspond to the dimensionless luminosity distance and amplification factor of lensed GW.

### Precision of lens reconstruction

Newly developed state-of-the-art lens modeling techniques and kinematic modeling methods have demonstrated their power to extract the information about lens mass distribution from high-quality imaging of strong lensing systems^28^. On the other hand, spectroscopic data for the central parts of lensing galaxies became available, which made it possible to assess central velocity dispersions inside the aperture. We assumed the uncertainty of the velocity dispersion at the level of 5% and the uncertainty of the Einstein radius measurements at the level of 1%. The assumed accuracy of the Einstein radius measurements is reasonable for the future LSST survey. One can expect that the high resolution imaging of LSST, with different stacking strategies for combining multiple exposures, making possible that a deeper stacked image could be obtained with the combination of individual exposures for each object^29^, will enable such precise Einstein radius measurements. The joint gravitational lensing and stellar-dynamical analysis of the SL2S lens sample^30^ has shown that it is feasible to determine the total mass-density slope inside the Einstein radius at the level of 5%. However, the supplementary information about time delays can reduce this uncertainty to 1% level as suggested by previous analysis of lensed quasars^31^. One can be optimistic in this respect, considering over a decade of efforts made by the H0LiCOW collaboration^32^ to develop techniques and gather data with sufficient constraining power, using time delays measured by the COSMOGRAIL collaboration^33^.

### Precision of time-delays

Precise time delays are the crucial point of our idea. As time delays Δ*t* between strongly lensed gravitational wave signals can be measured with an unprecedented accuracy of ~0.1 *s* from the detection pipeline^23^ or even by many orders of magnitude higher if the details of the waveform are analyzed, e.g. the moment of the final coalescence can be determined with ~10^−4^ *ms* accuracy. Let us stress, that the Fermat potential is of particular relevance for our analysis, due to its direct dependence on the logarithmic slope of the mass profile at the Einstein radius. Therefore, the uncertainty of the Fermat potential difference should be simulated from the lens mass profile and the Einstein radius uncertainties^32^. If the EM counterpart is detectable allowing for host galaxy image analysis and the measurement of central velocity dispersion of the lens, one can achieve the ~0.5% precision level of the Fermat potential difference provided the lensed host image quality is typical to the HST observations^23^. Finally, the mass distribution along the line of sight, i.e., LOS contamination might introduce 1% uncertainty of the lens potential recovery. This justifies the assumption of a few percent uncertainty on the Fermat potential measurements.

In order to demonstrate the performance of our method, we simulated a population of 10000 realistic strong lensing systems that could be observed by the Large Synoptic Survey Telescope (LSST)^34^, in which the lenses are modeled with the power-law mass distribution (*ρ*~*r*^−*γ*^). Details of the simulation are given in the Methods section. The main target of this work are lensed GW signals from NS-NS and BH-NS systems with high signal to noise ratio (SNR) and up to high redshifts. We performed Monte Carlo simulation to create a mock catalog of luminosity distances to the GW events that can be detected with *SNR* > 8 (for details see the Methods section). In particular, we refer to the third generation of interferometers – the “xylophone” configuration for the Einstein Telescope (ET)^35^ and the second generation technology for the Big Bang Observer (BBO)^36^. If the EM counterpart like a short gamma ray burst (SGRB) could be visible, this would facilitate identification of the host galaxy and measurement of the redshift. In addition, BBO’s angular resolution would be sufficient to uniquely identify the host galaxy for the majority of binaries, the redshifts of which could be obtained from a coordinated optical/infrared observing campaigns.

**Figure 1:**
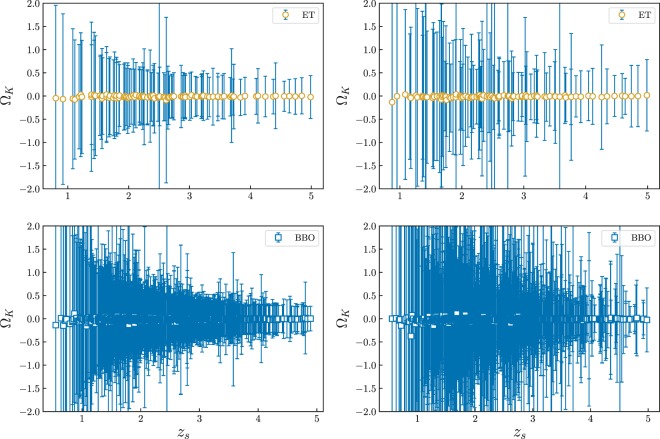
An example of the simulated measurements of the cosmic curvature from future observations of lensed GWs. We simulated 100 lensed GW signals detectable by the ET (upper panel) and 1000 signals detectable by the BBO (lower panel). Left and right panel respectively show the results with 10% and 50% uncertainty in the amplification factor measurements.

**Figure 2:**
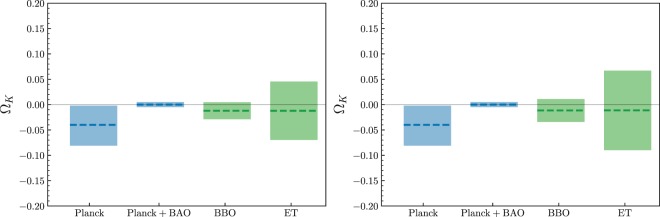
Statistical summary of simulated predictions of the Ω_*k*_ parameter measurements (inverse variance weighting) from future observations of lensed GWs. Left and right panel respectively show the results with 10% and 50% uncertainty in the amplification factor measurements. Predictions for the ET and the BBO are confronted with constraints achievable from the CMB and BAO measurements.

An example of the simulation results obtained from future ET and BBO survey is shown in Fig. 1, while the statistical constraints on the constant FLRW parameter Ω_*k*_ are presented in Fig. 2.

## Discussion

In light of the results presented above, the question arises: *Are these measurements sufficient enough to detect possible deviation from the Friedmann-Lematre-Robertson-Walker metric?* Considering relatively poor-precision measurements of Ω_*k*_(*z*_*l*_, *z*_*s*_) in the lower redshift region (*z* < 1), it is very difficult to achieve results competitive with other probes. However, the distributions of lensed GWs detectable by ET and BBO have tails reaching even *z* = 5. Therefore, we expect that ET with its potential of discovering a large number of lensed GWs will yield hundreds of measurements of cosmic curvature. Compared with ground-based GW detectors, space-borne detectors would provide order of magnitude bigger catalog of more precise Ω_*k*_ measurements. This would increase chance of finding significantly different Ω_*k*_(*z*_*l*_, *z*_*s*_) for different pairs (*z*_*l*_, *z*_*s*_) in the case when FLRW metric breaks down on some large scale. In principle, the function Ω_*k*_(*z*_*l*_, *z*_*s*_) can be reconstructed from observations, and the FLRW metric is ruled out if Ω_*k*_(*z*_*l*_, *z*_*s*_) is not constant^11–13^. However, one should note that, like all nonlinear functions of the measurements, the definition of Ω_*k*_(*z*_*l*_, *z*_*s*_) provided in Eq. (9) could provide a biased estimate of cosmic curvature due to the nonlinear propagation of measurement uncertainties. In any case, given the limited sample size of lensed GW events, we do not try to find Ω_*k*_ as a function of redshifts. Instead, we fit a constant Ω_*k*_ to the data and consider the accuracy of fit. The problem was previously recognized by^15^ and extensively discussed in^17^, with a heuristic suggestion that appropriate weighting on the Ω_*k*_ estimator can provide a substantially unbiased result. Therefore, in order to clarify and study the systematics and scatter in our results, we will make summary statistics in three ways: standard weighted mean, modified weighted mean, and median statistics. Such a procedure has been applied to the so-called *Om*(*z*) diagnostic defined with the expansion rate of the Universe^37,38^.

The effectiveness of of our method could be seen from discussion of the second question, that is: *Is it possible to achieve a stringent measurement of the present value of curvature density parameter?* The most straightforward and popular way of summarizing multiple measurements is inverse variance weighting:

3 $$\begin{array}{l}{\bar{{\rm{\Omega }}}}_{k}=\frac{\sum {({\rm{\Omega }}}_{k,i}/{\sigma }_{{{\rm{\Omega }}}_{k,i}}^{2})}{\sum 1/{\sigma }_{{{\rm{\Omega }}}_{k,i}}^{2}},\\ {\sigma }_{{\bar{{\rm{\Omega }}}}_{k}}^{2}=\frac{1}{\sum 1/{\sigma }_{{{\rm{\Omega }}}_{k,i}}^{2}},\end{array}$$

where ${\bar{{\rm{\Omega }}}}_{k}$ stands for the weighted mean of cosmic curvature and ${\sigma }_{{\bar{{\rm{\Omega }}}}_{k}}$ is its uncertainty. Let’s start from the first case by introducing an overall 10% uncertainty to the amplification factor measurement, concerning the estimation of luminosity distance to the lensed GW. The forecast for the ET is: Ω_*k*_ = −0.011 ± 0.057. This result illustrated in Fig. 2 is comparable to that derived from the current estimation of the cosmic curvature (${{\rm{\Omega }}}_{k}=-\,{0.040}_{-0.041}^{+0.038}$) from the *Planck* 2016 CMB data (the power spectra (TT, TE, EE+lowP))^1^. Such conclusion is also well consistent with the recent analysis of^39^, which discussed constraints on cosmological parameters in FLRW metric by combining the time delay distances from lensed GW signals, together with the co-moving distances obtained from a parametrized fitting approach with independent SNe Ia observations. In the case of the BBO providing more measurements of Ω_*k*_ with different redshift pairs, one can expect that cosmic curvature could be estimated with the precision of Ω_*k*_ = −0.012 ± 0.017. Admittedly, combination of CMB *Planck* and BAO data leads to a very high precision Ω_*k*_ = 0.000 ± 0.005 but this result pre-assumes the FLRW metric^1^. Such assumption can be tested empirically by comparing the spatial curvature determined from lensed GWs (detected by BBO) with that obtained from CMB + BAO data. In the second case, when the fractional uncertainty of the amplification factor measurement is assumed at the level of 50%, the resulting constraints on the cosmic curvature become Ω_*k*_ = −0.012 ± 0.073 for the ET and Ω_*k*_ = −0.010 ± 0.023 for the BBO. The statistical results for Ω_*k*_ is shown in Fig. 2. From this plot it is evident that, in the framework of the methodology proposed in this paper, the precision of derived cosmic curvature is sensitive to the adopted amplification factor measurements. This illustrates the importance of using auxiliary data to improve constraints on the amplification factor, especially the microlensing effect, with future more precise measurements for local image environments and more knowledge on AGN accretion model from astrophysics inputs^40^.

If one is to make summary statistics, one can do it in another two ways. Firstly, considering the multiple powers of multiple distances in the Ω_*k*_ estimation, different terms will prefer different weighting^15^. Following the procedure proposed by^17^, we apply purely empirical analysis to the simulated sample and determine what weighting is most successful in debiasing cosmic curvature. Our result show that appropriate weighting with $1/{\sigma }_{{{\rm{\Omega }}}_{k}}^{0.3}$ works well for Ω_*k*_ estimator. Proceeding this way, we have obtained Ω_*k*_ = −0.006 ± 0.092 for the ET and Ω_*k*_ = −0.005 ± 0.029 for the BBO, by introducing an overall 10% uncertainty to the amplification factor measurement. Secondly, it is well known that the weighted mean approach relies on several strong assumptions: a statistical independence of the data, no systematic effects, and a Gaussian distribution of the errors. Given the possible invalidity of the above assumptions (especially the Gaussianity of errors), another much more robust approach, the non-parametric median statistics is also applied in our analysis, without the need to assume anything about the error distribution. Such approach stems from the well known property that for any particular measurement, half of the data is expected to be higher and another half lower than the median^41,42^. Therefore, the probability that *n*-th observation out of the total number of *N* is higher than the median follows the binomial distribution: *P* = 2^−*N*^*N*!/[*n*!(*N* − *n*)!], which defines the 68.3% confidence intervals of the median^37,38^. In the framework of the median statistics, when the fractional uncertainty of the amplification factor measurement is assumed at the level of 10%, the resulting constraints on the cosmic curvature become ${{\rm{\Omega }}}_{k}=-\,{0.007}_{-0.020}^{+0.018}$ for the ET and ${{\rm{\Omega }}}_{k}=-{0.006}_{-0.018}^{+0.016}$ for the BBO. Therefore, one should pay careful attention to the bias induced by nonlinearity of the error propagation. For the current data sets of less than 1000 strong lens systems, this is of little real concern in the present case, since the scatter always dominates over the bias in this case^17^. If looking beyond this, one possible solution to this issue can also be found in^15^, which applied the *χ*^2^ statistics to fit constant Ω_*k*_ to 30 galatic-scale lenses from Sloan Lens ACS Survey. Their results showed the lenses’s goodness of fit could provide evidence for deviations from the FLRW metric. If the FLRW hypothesis is not rejected with reasonable value of *χ*^2^/*d*.*o*.*f*., the probability distribution of Ω_*k*_ could be directly obtained from the *χ*^2^ values. For next generation data sets of more than 1000 strong lens systems, as was pointed in^17^, appropriate summary statistics only provide a demonstration of principle that bias can be reduced. In this case, Monte Carlo simulations of the actual data characteristics should be employed for dealing with bias, which can be furthermore performed in an be iterative way: subtract the modeled bias for Ω_*k*_ = 0, estimate the new Ω_*k*_, and resimulate^17^.

To summarize, let us clarify some simplified assumptions underlying our method. For instance, in this paper, we adopt the geometrical optics approximation to derive the information of GW luminosity distances, which is valid in all the observational events of gravitational lensing of light. For the gravitational lensing of gravitational waves, the wavelength is long so that the geometrical optics approximation is not valid in some cases. As shown in^43^ and in a more detailed way in^44^, the wave optics should be used instead of the geometrical optics when the wavelength of the gravitational waves *λ* is longer than the Schwarzschild radius of the lens mass *M*_*L*_. More specifically, the diffraction effect is important for *M*_*L*_ ≤ 10^8^*M*_◉_(*f*/mHz)^43^. The wave effects become important for the lens mass 10–10^4^ *M*_◉_ and 10^5^–10^7^ *M*_◉_, which is determined by the ET band (10–10^4^ Hz) and the BBO band (10^−2^–1 Hz), respectively. Therefore, in our approach this effect does not significantly contribute to the scatter in the final results. We make a final comment that, in order to implement our method, dedicated observations including spectroscopic redshift measurements of the lens and the source, velocity dispersion of the lens, higher angular resolution imaging to measure the Einstein radius, and dedicated campaigns to measure time delays would be necessary. Obtaining these data for a large sample of strongly lensed GWs will require substantial follow-up efforts. Despite of these difficulties, the approach, introduced in this paper, might provide a new window to engage multiple measurements of more galactic-scale lensing systems where strongly lensed gravitational waves and their electromagnetic counterparts can be simultaneously detected. With a sample of lensed GWs, we could be optimistic about detecting possible deviation from the FLRW metric within our observational volume in the future. Such accurate measurements of the FLRW metric can become a milestone in precision GW cosmology.

## Methods

### Outline of the method

We model the lens with a power-law mass distribution (*ρ*~*r*^−*γ*^) motivated by several previous studies, which found that early-type galaxies are well described by power-law mass distributions in regions covered by the X-ray and lensing observations^45^, as well as the pixelated lens potential corrections applied to gravitational lenses^46^. Based on the combination of the mass *M*_*lens*_ inside the Einstein radius and the dynamical mass inside the aperture *θ*_*ap*_ projected to lens plane, the spherical Jeans equation^47^ enables to assess the distance ratio^48,49^

4 $$\frac{{d}_{ls}}{{d}_{s}}=\frac{{D}_{ls}^{A}}{{D}_{s}^{A}}=\frac{{\theta }_{E}}{4\pi }\frac{{c}^{2}}{{\sigma }_{ap}^{2}}{(\frac{{\theta }_{E}}{{\theta }_{ap}})}^{\gamma -2}f{(\gamma )}^{-1}$$

where *f*(*γ*) is a certain function of the radial mass profile slope^47^ and *σ*_*ap*_ is the luminosity averaged line-of-sight velocity dispersion of the lens inside the aperture. It is clear that *σ*_*ap*_, *θ*_*E*_, *θ*_*ap*_ and *γ* obtained from the observations enable one to measure the distance ratio *d*_*ls*_/*d*_*s*_.

Time delays between GW signals ***θ***_*i*_ and ***θ***_*j*_ depend on the “time-delay distance” (*D*_Δt_) and the lens mass distribution^50^

5 $${\rm{\Delta }}{t}_{i,j}=\frac{{D}_{{\rm{\Delta }}t}(1+{z}_{{\rm{l}}})}{c}{\rm{\Delta }}{\varphi }_{i,j},$$

where Δ*ϕ*_*i*,*j*_ = [(***θ***_*i*_ − ***β***)^2^/2 − *ψ*(***θ***_*i*_) − (***θ***_*j*_ − ***β***)^2^/2 + *ψ*(***θ***_*j*_)] is the Fermat potential difference determined by the lens mass distribution and the source position ***β***, *ψ* denotes two-dimensional lensing potential determined by the Poisson equation ∇^2^*ψ* = 2*κ*, where *κ* is dimensionless surface mass density (convergence) of the lens. Hence, the time-delay distance is

6 $${D}_{{\rm{\Delta }}t}\equiv \frac{{D}_{{\rm{l}}}^{A}{D}_{{\rm{s}}}^{A}}{{D}_{{\rm{ls}}}^{A}}=\frac{c}{1+{z}_{l}}\frac{{\rm{\Delta }}{t}_{i,j}}{{\rm{\Delta }}{\varphi }_{i,j}}.$$

Therefore, the combined observations of strongly lensed EM and GW signals coming from the same source will provide the comoving distance to the lensing system

7 $${D}_{l}=(1+{z}_{l}){D}_{{\rm{\Delta }}t}\frac{{D}_{ls}^{A}}{{D}_{s}^{A}}.$$

Inspiralling compact binary systems act as self calibrating standard sirens providing the luminosity distance to the source. However, there is a degeneracy between GW inferred luminosity distance *D*^*L*^ and lensing magnification $A \sim \sqrt{\mu }/{D}^{L}$. Detailed analysis of the images of host galaxy and accompanying EM counterpart, can provide a measurement of the magnification of images and break down the degeneracy in GW based inference. It can be best illustrated with the singular isothermal sphere (SIS) lens, when two images *x*_±_ = *y* ± 1 are formed (*x* = *θ*/*θ*_*E*_, *y* = *β*/*θ*_*E*_) with flux magnification *μ*_±_ = 1/*y* ± 1. From the flux ratio one can infer the source position $y=\frac{1-{F}_{-}/{F}_{+}}{1+{F}_{-}/{F}_{+}}$. Once the source position is known, one can quantify the amplification of each image and estimate the luminosity distance *D*^*L*^ from GW waveforms. Therefore, the transverse comoving distance from the observer to the source could be derived as

8 $${D}_{s}=\frac{1}{1+{z}_{s}}{D}_{s}^{L}$$

provided the GW source’s redshift is known from the identification of an EM counterpart and its host galaxy. Combining the above analysis with Eq. (2), one can see that the function Ω_*k*_(*z*_*l*_, *z*_*s*_) can be directly obtained.

### Simulated lenses

We conservatively consider only elliptical galaxies, which contribute ~80% to the total lensing probability^51^. It was found that grid-based lens potential corrections from power-law models were only 2%^46^, further justifying the use of a simple power-law model to describe the mass distribution even for complicated lenses. In particular, various studies have shown that the power-law profile provides an accurate description of lens galaxies, out to *z*_*l*_~1, which are observed in number of large surveys (see ref.^49^ for the lens redshift distribution in SLACS, BELLS, SL2S and LSD). At this point, one should clarify the issue whether the power-law model is valid for high-redshift lenses, since it was suggested that LSST is also capable of discovering higher redshift lenses than currently known^34^ (the lens redshift of our simulated sample may reach to *z*_*l*_~1.9). However, in our analysis, the magnitude of uncertainty generated by such issue might be overestimated: on the one hand, the well-known modified Schechter function^52^ already predicts no significant lens population at high redshift; on the other hand, although the high-redshift galaxies with measured velocity dispersions is small, no significant dependence of the slope parameter *γ* on redshift (in the framework of power-law model) has been found so far based on lensing and dynamical analysis^53^. In order to ascertain similarity between our simulations and the real world, we assumed velocity dispersion of lenses as *σ*_*ap*_ = 210 ± 50 km/s and lens redshift distribution with the median value of *z*_*l*_ = 0.8, which is consistent with the properties of the LSD sample. We are thus confident that the simulated population of lenses is a good representation of what the future surveys might yield, considering the similarity of the redshift distribution of discoverable lenses in forthcoming LSST survey with that found in^54^.

### Lensed GW mock catalog

One can expect that both ET and BBO should register a considerable catalog of such events during a few years of successful operation: from 5~10 years’ accumulated data ~100 lensed GW events will be detected by three nested ET interferometers in the redshift range *z* ≤ 5.00^23,25^. The BBO, a proposed space-based GW detector, would possibly detect ~1000 lensed GW events from 10^6^ compact-star binaries^36^. Construction of the mock catalog proceeded along the following steps. The lensing rate strongly depends on the estimate of the GW event rate, which depends on the estimate of the merger rate of double compact objects (DCO). In this paper, we have adopted the conservative SFR function from^55^ and taken the data from the website *http:* www.syntheticuniverse.org, the so-called “rest frame rates” in cosmological scenario. Concerning gravitational lensing, the velocity dispersion distribution in the population of early-type galaxies was modelled as modified Schechter function with parameters from the SDSS DR3 data^56^. Based on the intrinsic merger rate of DCO calibrated by strong lensing effects^26^, we obtained the differential rates of lensed GW events as a function of *z*_*s*_, which furthermore constituted the sampling distribution of lensed GWs.

For each lensed GW event, the mass of the neutron star, the mass of the black hole, and the position angle *θ* are randomly sampled in the three parameter intervals: [1, 2] *M*_◉_, [3, 10] *M*_◉_, and [0, *π*]^16^. It was well acknowledged that luminosity distances can be inferred from the waveform of GW signals from chirping binaries. Different sources of uncertainties are included in our simulation of luminosity distance ${D}_{s}^{L}$. Firstly, for ET, the combined SNR for the network of three independent interferometers is defined by the inner product^57^ of the Fourier transform *H*(*f*) of the time domain waveform *h*(*t*), which not only confirms the detection of GW with *ρ*_*net*_ > 8.0, but also contributes to the error on the luminosity distance as ${\sigma }_{inst}\simeq 2{D}_{s}^{L}/{\rho }_{net}$. In our calculation, the upper cutoff frequency is dictated by the last stable orbit *f*_*upper*_ = 2*f*_*LSO*_, while the lower cutoff frequency is taken as *f*_*lower*_ = 1*Hz*. The BBO, which is fundamentally self-calibrating, would determine the luminosity distance to each binary to 1% accuracy. More specifically, we assume that the distance measurement errors due to detector noise for each individual binary are those shown in^36^. Secondly, following the strategy described by^58^, weak lensing has been estimated as a major source of error on ${D}_{s}^{L}(z)$ for standard sirens. For the ET we estimated the uncertainty from weak lensing according to the fitting formula^59^ ${\sigma }_{lens}/{D}_{s}^{L}=0.05z$. For the BBO, since the systematic distance errors arising from the detector itself will also be negligible, the uncertainty of the true distance to the binary system will be dominated by the effects of weak lensing^36^, parameterized as ${\sigma }_{lens}/{D}_{s}^{L}=0.044z$.

In the case of strongly lensed GWs, the luminosity distance could be estimated with a better knowledge of the lensing amplification factor for the GW signal. More specifically, the lensing amplification factor (of the GW signal) could be derived from the lensing magnification (of the EM observation), with the latter determined by solving the lens equation using *glafic*^60^. Therefore, the uncertainty of *F* is related to that of the lens mass profile and the Einstein radius, which can be derived from high-quality imaging observations^61^. All lensed GW signals (images) will be used to determine luminosity distances and corresponding lensing amplification factors. Note that the lensing magnification is dependent on the lens model, which is the only way to determine the magnification for almost all strong lensing systems such as the quasar-galaxy and galaxy-galaxy systems^51,60^. We emphasize that only for the strongly lensed standard candles, SNe Ia, the magnification can be derived from comparing the observed brightness to other SNe Ia within a narrow redshift range^62^, such measurement of the lensing magnification is independent of any assumptions on cosmology and lens model. Finally, the influence of the microlensing (ML) effect generated by stars in lensing galaxy should be considered in the estimation of amplification factors. Due to the inclination of the finite AGN accretion disc and the differential magnification of the coherent temperature fluctuations, the microlensing by the stars can lead to changes in the actual magnification of the lensed EM signal (e.g. flux-ratio anomalies). The problem was recently recognized concerning galactic-scale strong lensing systems with SNe Ia as background sources, with a heuristic suggestion that adding an additional uncertainty ~0.70 mag to lensed SNe Ia^63^. However, there still exist a lot of uncertain inputs for the microlensing amplification factor priors, concerning thehost galaxy or accompanying EM counterpart of GW. On the one hand, it has been recently proved that the adopted local environments for images and mass function for the stars, which can generate different local convergency, shear and star proportion, could systematically bias the magnification map^64^. On the other hand, the inclination, position angle, especially the size of the accretion disk, as well as the relative motion of the source, would also bring uncertainties^65^. Recent discussion on this issue can be found in^40^. Taking the above factors into consideration, in our simulated data we choose to respectively introduce 20% and 100% uncertainty to the magnification measurements, which corresponds to 10% and 50% uncertainty to the amplification factor (*δF* = 10%, 50%), and quantify its effect on the cosmic curvature estimation.

### Cosmic curvature estimation

Now one is able to determine the cosmic curvature as

9 $${{\rm{\Omega }}}_{k}({z}_{l},{z}_{s})=\frac{{d}_{l}^{4}+{d}_{s}^{4}+{d}_{ls}^{4}-2{d}_{l}^{2}{d}_{s}^{2}-2{d}_{l}^{2}{d}_{ls}^{2}-2{d}_{s}^{2}{d}_{ls}^{2}}{4{d}_{l}^{2}{d}_{s}^{2}{d}_{ls}^{2}}$$

It should be noted that, when using the distance information of the lensing system itself, one does expect errors from *d*_*l*_ to be covariant with *d*_*ls*_ and *d*_*s*_. In particular^17^, addressed the problem of distance correlation and proposed using a different combination of distances (*d*_Δ*t*_, *d*_*l*_ and *d*_*s*_) to get Ω_*k*_. The dimensionless time-delay distance is related to the time-delay distance as *D*_Δ*t*_ = *cd*_Δ*t*_/*H*_0_. Because distances measured at widely separated redshifts should be mostly uncorrelated, they proposed that *d*_*l*_ and *d*_*s*_ could be derived from the reconstruction of the distance-redshift relation in a model independent manner, concerning the available standardized candle (Type Ia supernovae) or standard ruler (BAO) data. Putting together Eqs (4–9), one can rewrite the cosmic curvature in terms of measurable distances

10 $$\begin{array}{c}{{\rm{\Omega }}}_{k}({z}_{l},{z}_{s})=\frac{1}{4}\frac{{(1+{z}_{l})}^{2}{d}_{{\rm{\Delta }}t}^{2}}{{d}_{s}^{4}}+\frac{1}{4}\frac{{(1+{z}_{l})}^{2}}{{d}_{{\rm{\Delta }}t}^{2}{({d}_{ls}/{d}_{s})}^{4}}+\frac{1}{4}\frac{1}{{(1+{z}_{l})}^{2}{d}_{{\rm{\Delta }}t}^{2}}\\ \,-\frac{1}{2}\frac{1}{{({d}_{ls}/{d}_{s})}^{2}{d}_{s}^{2}}-\frac{1}{2}\frac{1}{{d}_{s}^{2}}-\frac{1}{2}\frac{1}{{(1+{z}_{l})}^{2}{d}_{{\rm{\Delta }}t}^{2}{({d}_{ls}/{d}_{s})}^{2}}\end{array}$$

which will be the central equation in our analysis. In order to carry out the curvature estimation, we need a measurement of *d*_*ls*_/*d*_*s*_ from image configuration, lens modeling and dynamics, *d*_Δ*t*_ from a strongly lensed time delay system, and *d*_*s*_ from the lensed standard siren. In this paper, instead of propagating distance uncertainties, we turn to the other effective solution to this problem, i.e., the uncertainty on the determination of the curvature is given in terms of the measurement uncertainties on the observables characterizing the lens mass profile (*γ*, *θ*_*E*_, *θ*_*ap*_), time delays (Δ*t*), Fermat potential difference due to lens and line-of-sight effect (Δ*ψ*, Δ*ψ* (LOS)), the luminosity distance to the source (${d}_{s}^{L}$), and the amplification factor (*F* with microlensing effect). Note that in the formalism presented in this paper, the mass profile of the lensing galaxy is described by *γ* and *θ*_*E*_, which is related to the lens potential difference (Δ*ψ*). The amplification factor also depends on the lens model, and thus the mass profile of the lensing galaxy. However, considering the uncertain influence of the microlensing (ML) effect, in our analysis we choose to introduce an overall 10% and 50% uncertainty to the amplification factor measurements. The uncertainties in these parameters are presumably covariant, which indicates that per-parameter uncertainties treated independently would be expected to underestimate propagated uncertainties. After considering such covariance, the uncorrelated measurement uncertainties on the observables of *γ*, *θ*_*E*_, *θ*_*ap*_, Δ*t*, Δ*ψ* (LOS), ${d}_{s}^{L}$, and *F* will be projected on the determination of Ω_*k*_ (Eq. (10))

11 $$\delta {{\rm{\Omega }}}_{k}({z}_{l},{z}_{s}) \sim (\delta \gamma ,\delta {\theta }_{E},\delta {\theta }_{ap},\delta ({\rm{\Delta }}t),\delta ({\rm{\Delta }}\psi (LOS)),\delta {d}_{s}^{L},\delta F).$$

More specifically, given the nonlinear combination of observables that goes into the curvature estimation, we use Monte-Carlo simulation to project uncertainties onto the final uncertainty of Ω_*k*_(*z*_*l*_, *z*_*s*_).
